# The choice of a neoadjuvant chemotherapy cycle for breast cancer has significance in clinical practice: results from a population-based, real world study

**DOI:** 10.20892/j.issn.2095-3941.2020.0800

**Published:** 2021-10-12

**Authors:** Litong Yao, Zhiyuan Pang, Mozhi Wang, Mengshen Wang, Xiangyu Sun, Mingke Cui, Yanfu Zheng, Xinyan Li, Haoran Dong, Qiang Zhang, Yingying Xu

**Affiliations:** 1 Department of Breast Surgery, the First Affiliated Hospital of China Medical University, Shenyang 110001, China; 2 Department of Breast Surgery, Cancer Hospital of China Medical University, Liaoning Cancer Hospital & Institute, Shenyang 110042, China

**Keywords:** Breast cancer, neoadjuvant chemotherapy, treatment cycles, real world analysis, survival analysis

## Abstract

**Objective:**

Neoadjuvant chemotherapy (NAC) is currently used in both early stage and locally advanced breast cancers. The survival benefits of standard *vs.* non-standard NAC cycles are still unclear. This study aimed to investigate the relationship between NAC cycles and survival based on real world data.

**Methods:**

We identified patients diagnosed with invasive primary breast cancers who underwent NAC followed by surgery. Patients who received at least 4 NAC cycles were defined as having received standard cycles, while patients who received less than 4 NAC cycles were defined as having received non-standard cycles. Kaplan-Meier curves and Cox proportional hazard models were used to estimate the disease-free survival (DFS) and overall survival (OS).

**Results:**

Of the 1,024 included patients, 700 patients received standard NAC cycles and 324 patients received non-standard NAC cycles. The DFS estimates were 87.1% and 81.0% (*P* = 0.007) and the OS estimates were 90.0% and 82.6% (*P* = 0.001) in the standard and non-standard groups, respectively. Using multivariate analyses, patients treated with standard NAC cycles showed significant survival benefits in both DFS [hazard ratio (HR): 0.62, 95% confidence interval (CI): 0.44–0.88] and OS (HR: 0.54, 95% CI: 0.37–0.79). Using stratified analyses, standard NAC cycles were associated with improved DFS (HR: 0.59, 95% CI: 0.36–0.96) and OS (HR: 0.49, 95% CI: 0.28–0.86) in the HER2 positive group. Similar DFS (HR: 0.50, 95% CI: 0.25–0.98) and OS (HR: 0.45, 95% CI: 0.22–0.91) benefits were shown for the triple negative group.

**Conclusions:**

Standard NAC cycles were associated with a significant survival benefit, especially in patients with HER2 positive or triple negative breast cancer.

## Introduction

Breast cancer is one of the most common causes of cancer-related death among women worldwide, and it is the most frequently diagnosed type of cancer in women^1^. Neoadjuvant chemotherapy (NAC) is defined as chemotherapy that is administered before locoregional treatment, such as surgery and/or irradiation. NAC is considered standard care for patients with locally advanced breast cancers, and it has also become an effective treatment option for patients with early stage breast cancers^2,3^. Recently, the use of NAC has been more widely applied. The neoadjuvant approach allows for the rapid assessment of treatment efficacy, and a response-guided neoadjuvant protocol can lead to modifications of both neoadjuvant and adjuvant chemotherapies^4,5^. The presence or absence of residual tumors can guide the selection of escalation or de-escalation strategies for subsequent adjuvant chemotherapy. Adding capecitabine or trastuzumab emtansine (T-DM1) to the original adjuvant therapy showed evidence of a survival advantage in patients with residual invasive disease after preoperative systemic therapy, in the CREATE-X and KATHERINE trials^6,7^. Conversely, the WSG-ADAPT-TN trial indicated that patients with a pathological complete response (pCR) might not require adjuvant chemotherapy^8^. Consequently, patients with more aggressive subtypes of breast cancer, including human epidermal growth factor receptor-2 (HER2) positive and triple negative breast cancer (TNBC), are candidates for NAC according to the 2020 update of the National Comprehensive Cancer Network (NCCN) guidelines.

The National Surgical Adjuvant Breast and Bowel Project (Protocols B-18 and B-27) showed that NAC had the same efficacy as adjuvant chemotherapy for patients with breast cancer, if similar regimens were used^9,10^. Standard NAC regimens are therefore designed to closely follow adjuvant regimens that require at least 4–6 cycles over 6–12 months^11^. However, standard cycles may be halted during the therapy period if the tumor size improves sufficiently, as the historic objective of NAC is to render inoperable cancers operable or to shrink tumors to facilitate breast conservation^12^. Discontinuation of NAC may also occur if patients are intolerant to the regimen, unwilling to continue the therapy, or if satisfactory outcomes are not observed. The unfinished portions of the NAC regimen are usually added to the patients’ adjuvant therapy following surgery. However, the differences in outcomes between these 2 approaches are still unclear. Here, we conducted a population-based real world study comparing survival outcomes in patients with breast cancer who underwent standard or non-standard cycles of NAC. We further analyzed various subgroups of patients according to their biological subtypes, pathological responses, or clinical responses to provide guidelines for personalized precision therapy.

## Materials and methods

### Study population

A real world cohort study was conducted including patients diagnosed with primary invasive breast cancers who were treated with NAC at the Department of Breast Surgery at the First Affiliated Hospital of China Medical University and the Cancer Hospital of China Medical University between February 2012 and September 2017. All patients received at least 1 cycle of neoadjuvant treatment followed by surgery, with adjuvant radiotherapy, chemotherapy, and endocrine therapy provided when indicated. The exclusion criteria included patients receiving any type of treatment prior to NAC, patients suffering from progression and metastatic diseases prior to surgery, and patients with previous or synchronous invasive or *in situ* breast cancers, bilateral breast cancers, male breast cancers, or inflammatory breast cancers. All patients underwent a structured postoperative follow-up, with regular clinical visits and yearly imaging. Patients were categorized according to the number of NAC cycles. Standard NAC cycles were defined as treatment with at least 4 cycles of preoperative chemotherapy, and non-standard NAC cycles were defined as treatment with less than 4 NAC cycles followed by adjuvant therapy.

### Measurements of clinicopathological features

The clinicopathological features included age at diagnosis, menopausal status, body mass index (BMI), clinical tumor grade, estrogen receptor (ER) status, progesterone receptor (PgR) status, HER2 status, Ki67 index, number of NAC cycles, post-NAC pathological node stage, pathological response, clinical response, types of surgery and adjuvant therapies, and chemotherapy strategies. ER status, PgR status, HER2 status, and the Ki67 index were independently evaluated by 2 experienced pathologists. Immunohistochemistry (IHC) analysis was performed on formalin-fixed, paraffin-embedded tissue sections following standard procedures for breast tumor specimens obtained from core needle biopsies and surgical resections. Tumor biological subtype was categorized as hormone receptor positive (HR, ER positive, and/or PgR positive), HER2 positive (regardless of ER and PgR status), or the TNBC. The Ki67 index was divided into high and low expression groups with a boundary of 20%. The pCR was defined as the absence of invasive carcinomas in both the breast and axillary nodes, irrespective of ductal carcinoma *in situ*, on pathological review of surgical specimens following NAC (ypT0/is, ypN0). The clinical response evaluation was based on the Response Evaluation Criteria in Solid Tumors (RECIST) v1.1^13^. Partial response (PR) was defined as a greater than 30% reduction in the longest diameter of the tumor from its initial size. Progressive disease (PD) was defined as a greater than 20% increase in the longest diameter of the tumor from the smallest value recorded, an absolute increase of at least 5 mm, the appearance of new lesions, or the unequivocal progression of nontarget lesions. Stable disease (SD) was defined as meeting neither PR nor PD criteria.

### Statistical analysis

All statistical analyses were performed using SPSS statistical software for Windows, version 23 (SPSS, Chicago, IL, USA). Disease-free survival (DFS) was calculated from the date of surgery to the occurrence of the first event (locoregional relapse, distant relapse, or death). Survival was determined at the end of follow-up for patients who were alive and had not experienced an event by the cut-off date. Overall survival (OS) was calculated as the interval from surgery to death, and patients who were alive on the cut-off date were censored at the last follow-up date. Using univariate analyses, continuous variables were compared using one-way ANOVA, and categorical variables were measured using the chi-square and Fisher’s exact tests. Survival was visualized using the Kaplan-Meier method and differences between the survival curves of different groups were analyzed using a log-rank test with a log-rank *P* < 0.05 defined as significant. Subgroup analysis was assessed according to biological subtype, pathological response, and clinical response. Using multivariate analysis, the Cox proportional hazard regression model was used to estimate the relationship between NAC cycles and survival after adjusting for potential confounders. Hazard ratio (HR) and 95% confidence interval (CI) were reported, with a 2-tailed *P* value < 0.05 considered as denoting significant differences.

## Results

A total of 1,024 patients who fulfilled the study criteria at the Department of Breast Surgery at the First Affiliated Hospital of China Medical University and Cancer Hospital of China Medical University were included in our study. Patients’ clinicopathological characteristics stratified by NAC cycles are shown in **Table 1**. The mean age of the included patients was 51 years (range, 25–76 years). Overall, 700 patients underwent standard NAC regimens, and 324 patients underwent non-standard NAC regimens. A total of 34.3% (*N* = 351) of the patients had HR positive/HER2 negative tumors, 48.3% (*N* = 495) of the patients had HER2 positive tumors, and 17.4% (*N* = 178) of the patients had triple negative tumors. A total of 116 patients (11.3%) achieved pCR and 908 patients (88.7%) had residual invasive disease in the breast and/or axillary lymph nodes. The univariate analysis revealed that the number of NAC cycles, the clinical tumor stage, ER status, PgR status, the biological subtype, post-NAC pathological node stage, pathological response, clinical response, and the use of adjuvant radiotherapy were all significantly correlated with the prognosis, as shown in **Table 2**.

**Table 1:** Patient characteristics by neoadjuvant chemotherapy cycles

Characteristics	Total	Standard cycles	Non-standard cycles	*P*
	*n* = 1,024	*n* = 700 (68.4%)	*n* = 324 (31.6%)	
Age at diagnosis (years)	51.01 ± 9.43	50.51 ± 9.58	52.10 ± 9.02	0.012*
Menopausal status				0.699
Premenopausal	296 (28.9%)	208 (29.7%)	88 (27.2%)	
Postmenopausal	434 (42.4%)	294 (42.0%)	140 (43.2%)	
Unknown	294 (28.7%)	198 (28.3%)	96 (29.6%)	
BMI				0.244
Underweight (< 18.5)	29 (2.8%)	16 (2.3%)	13 (4.0%)	
Normal (18.5–24.9)	644 (62.9%)	435 (62.1%)	209 (64.5%)	
Overweight (25–29.9)	310 (30.3%)	222 (31.7%)	88 (27.2%)	
Obese (> 30)	41 (4.0%)	27 (3.9%)	14 (4.3%)	
Clinical tumor stage				0.051
T1	111 (10.8%)	65 (9.3%)	46 (14.2%)	
T2	737 (72.0%)	509 (72.7%)	228 (70.4%)	
T3/T4	176 (17.2%)	126 (18.0%)	50 (15.4%)	
ER status				0.780
Negative	430 (42.0%)	296 (42.3%)	134 (41.4%)	
Positive	594 (58.0%)	404 (57.7%)	190 (58.6%)	
PgR status				0.449
Negative	514 (50.2%)	357 (51.0%)	157 (48.5%)	
Positive	510 (49.8%)	343 (49.0%)	167 (51.5%)	
HER2 status				0.650
Negative	529 (51.7%)	365 (52.1%)	164 (50.6%)	
Positive	495 (48.3%)	335 (47.9%)	160 (49.4%)	
Biological subtype				0.878
HR positive/HER2 negative	351 (34.3%)	241 (34.4%)	110 (34.0%)	
HER2 positive	495 (48.3%)	335 (47.9%)	160 (49.4%)	
Triple negative	178 (17.4%)	124 (17.7%)	54 (16.7%)	
Histological grade				0.179
1	15 (1.5%)	10 (1.4%)	5 (1.5%)	
2	521 (50.9%)	360 (51.4%)	161 (49.7%)	
3	81 (7.9%)	63 (9.0%)	18 (5.6%)	
Unknown	407 (39.7%)	267 (38.1%)	140 (43.2%)	
Baseline Ki67				0.026*
≤ 20%	297 (29.0%)	188 (26.9%)	109 (33.6%)	
> 20%	727 (71.0%)	512 (73.1%)	215 (66.4%)	
Post-neoadjuvant pathological node stage				0.491
ypN0	427 (41.7%)	300 (42.9%)	127 (39.2%)	
ypN1	287 (28.0%)	190 (27.1%)	97 (29.9%)	
ypN2	174 (17.0%)	122 (17.4%)	52 (16.0%)	
ypN3	136 (13.3%)	88 (12.6%)	48 (14.8%)	
Pathological response				< 0.0001*
pCR	116 (11.3%)	99 (14.1%)	17 (5.2%)	
Non-pCR	908 (88.7%)	601 (85.9%)	307 (94.8%)	
Clinical response				< 0.0001*
PR	584 (57.0%)	426 (60.9%)	158 (48.8%)	
SD	440 (43.0%)	274 (39.1%)	166 (51.2%)	
Type of surgery				0.548
BCS	47 (4.6%)	34 (4.9%)	13 (4.0%)	
Mastectomy	977 (95.4%)	666 (95.1%)	311 (96.0%)	
Adjuvant radiotherapy				0.375
Yes	624 (60.9%)	433 (61.9%)	191 (59.0%)	
No	400 (39.1%)	267 (38.1%)	133 (41.0%)	
Adjuvant endocrine therapy				0.326
Yes	598 (58.4%)	416 (59.4%)	182 (56.2%)	
No	426 (41.6%)	284 (40.6%)	142 (43.8%)	
Anti-HER2 targeted therapy				0.945
Yes	482 (47.1%)	330 (47.1%)	152 (46.9%)	
No	542 (52.9%)	370 (52.9%)	172 (53.1%)	
Chemotherapy strategies				0.389
Anthracycline- and taxane-based	715 (69.8%)	482 (68.9%)	233 (71.9%)	
Anthracycline-based only	206 (20.1%)	149 (21.3%)	57 (17.6%)	
Taxane-based only	103 (10.1%)	69 (9.9%)	34 (10.5%)	

*P < 0.05 was considered statistically significant.

BMI, body mass index; ER, estrogen receptor; PgR, progesterone receptor; HER2, human epidermal growth factor receptor-2; HR, hormone receptor; pCR, pathological complete response; PR, partial response; SD, stable disease; BCS, breast-conservation surgery.

**Table 2:** Univariate analysis for disease-free and overall survivals

Characteristics	Total (*n* = 1,024)	DFS		OS	
		Events (*n* = 136)	*P*	Events (*n* = 111)	*P*
Age at diagnosis (years)	51.01 ± 9.43	51.23 ± 9.38	0.772	50.88 ± 9.10	0.881
Menopausal status			0.648		0.882
Premenopausal	296	39		34	
Postmenopausal	434	62		47	
Unknown	294	35		30	
BMI			0.329		0.506
Underweight (< 18.5)	29	5		3	
Normal (18.5–24.9)	644	76		63	
Overweight (25–29.9)	310	48		39	
Obese (> 30)	41	7		6	
Clinical tumor stage			0.002*		0.001*
T1	111	13		11	
T2	737	85		67	
T3/T4	176	38		33	
ER status			< 0.0001*		< 0.0001*
Negative	430	76		66	
Positive	594	60		45	
PgR status			< 0.0001*		< 0.0001*
Negative	514	88		77	
Positive	510	48		34	
HER2 status			0.817		0.593
Negative	529	69		60	
Positive	495	67		51	
Biological subtype			0.006*		0.002*
HR positive/HER2 negative	351	34		28	
HER2 positive	495	67		51	
Triple negative	178	35		32	
Histological grade			0.890		0.849
1	15	2		2	
2	521	69		55	
3	81	13		11	
Unknown	407	52		43	
Baseline Ki67			0.191		0.966
≤ 20%	297	33		32	
> 20%	727	103		79	
NAC cycles			0.002*		< 0.0001*
Non-standard cycles	324	59		52	
Standard cycles	700	77		59	
Post-neoadjuvant pathological node stage			< 0.0001*		< 0.0001*
ypN0	427	32		26	
ypN1	287	33		26	
ypN2	174	40		32	
ypN3	136	31		27	
Pathological response			0.002*		0.007*
pCR	116	5		4	
Non-pCR	908	131		107	
Clinical response			0.007*		0.007*
PR	584	63		50	
SD	440	73		61	
Type of surgery			0.154		0.314
BCS	47	3		3	
Mastectomy	977	133		108	
Adjuvant radiotherapy			< 0.0001*		< 0.0001*
Yes	624	110		90	
No	400	26		21	
Adjuvant endocrine therapy			0.504		0.810
Yes	598	83		66	
No	426	53		45	
Anti-HER2 targeted therapy			0.267		0.208
Yes	482	58		46	
No	542	78		65	
Chemotherapy strategies			0.594		0.994
Anthracycline- and taxane-based	715	92		78	
Anthracycline-based only	206	27		22	
Taxane-based only	103	17		11	

*P < 0.05 was considered statistically significant.

BMI, body mass index; ER, estrogen receptor; PgR, progesterone receptor; HER2, human epidermal growth factor receptor-2; HR, hormone receptor; pCR, pathological complete response; PR, partial response; SD, stable disease; BCS, breast-conservation surgery.

**Figure 1:**
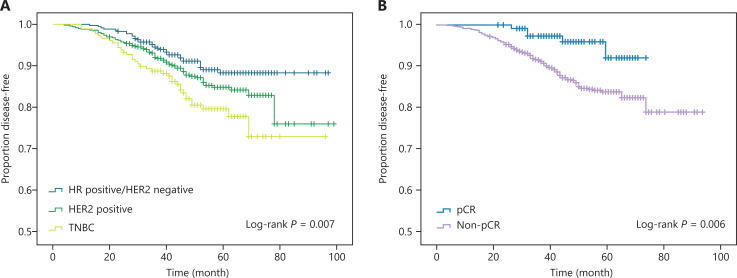
The Kaplan-Meier method estimated disease-free survival by biological subtypes (A) and pathological responses (B).

We observed similar results for DFS and OS, and we provided the OS curve in the Supplemental Digital Content section. **Figure 1 and Supplementary Figure S1** illustrate the DFS and OS according to the different biological subtypes and pathological responses. Kaplan-Meier curves showed significantly improved survival for patients with HR positive subtypes, followed by those with HER2 positive and triple negative subtypes [5-year DFS: 88.3%, 84.8%, and 79.6%, *P* = 0.007 (**Figure 1A**) and 5-year OS: 90.2%, 88.1%, and 80.8%, *P* = 0.002 (**Supplementary Figure S1A**)]. Patients who achieved pCR had a better DFS (*P* = 0.006) (**Figure 1B**) and OS (*P* = 0.013) (**Supplementary Figure S1B**). The associations between pathological responses and long-term outcomes according to tumor biological subtypes are shown in **Supplementary Table S1**. The pCR was significantly associated with greater survival in the triple negative subgroup than in the other groups (DFS: *P* = 0.031 and OS: *P* = 0.043); however, no significant correlation between pCR and survival was found among patients with HR positive or HER2 positive tumors.

**Figure 2:**
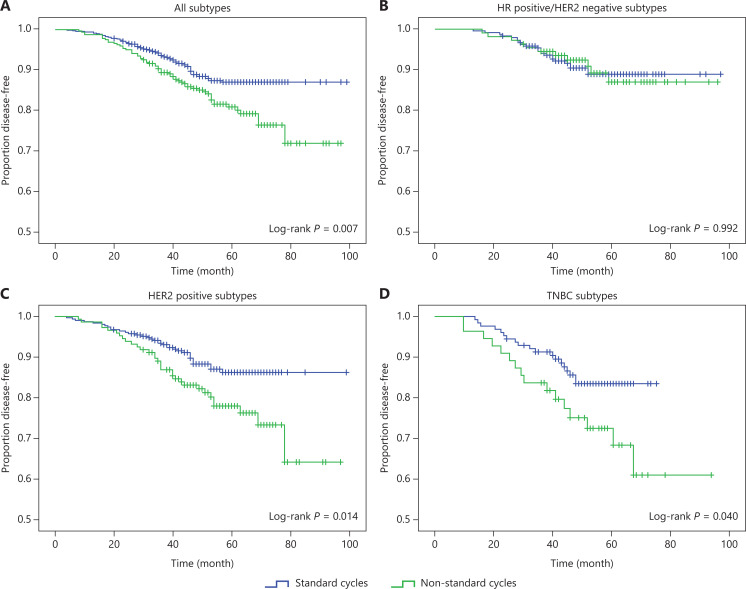
The Kaplan-Meier method estimated disease-free survival by standard and non-standard neoadjuvant chemotherapy cycles in overall subtypes (A), HR positive/HER2 negative subtypes (B), HER2 positive subtypes (C), and triple negative subtypes (D).

Kaplan-Meier curves showed that survival differences between patients who received standard NAC cycles and those receiving non-standard NAC cycles (**Figure 2 and Supplementary Figure S2**). For all patients, the number of NAC cycles was significantly associated with the DFS, with a 5-year DFS of 87.1% in patients receiving standard NAC cycles and 81.0% in patients receiving non-standard NAC cycles (*P* = 0.007) (**Figure 2A**). Similar outcomes were seen for OS (90.0% *vs*. 82.6%, *P* = 0.001) (**Supplementary Figure S2A**). Subgroup analysis of patients with HR positive/HER2 negative subtypes revealed no significant impact of standard NAC cycles on the DFS (*P* = 0.992) (**Figure 2B**) or OS (*P* = 0.71) (**Supplementary Figure S2B**). For patients with HER2 positive tumors, the 5-year DFS estimates were 87.4% in patients receiving standard NAC cycles and 79.8% in patients receiving non-standard NAC cycles (*P* = 0.014) (**Figure 2C**), and the 5-year OS percentages were 91.1% and 82.5% (*P* = 0.004) (**Supplementary Figure S2C**), respectively. For patients with TNBC, 5-year DFS estimates were 83.1% and 71.9% (*P* = 0.04) (**Figure 2D**), and 5-year OS percentages were 84.9% and 71.9% (*P* = 0.032) (**Supplementary Figure S2D**), in patients receiving standard and non-standard NAC cycles, respectively.

Using multivariate analysis, after adjusting for age at diagnosis, clinical tumor stage, biological subtype, and post-NAC pathological node stage, standard NAC cycles were associated with a better DFS (HR: 0.62, 95% CI: 0.44–0.88, *P* = 0.007) and OS (HR: 0.54, 95% CI: 0.37–0.79, *P* = 0.001) than the DFS and OS observed after treatment with non-standard NAC cycles (**Supplementary Table S2 and Table 3**). Standard cycles of NAC were associated with better DFS and OS than non-standard cycles after analyzing stratified multivariable models according to biological subtypes. The HR of patients with HER2 positive tumors was 0.59 (95% CI: 0.36–0.96, *P* = 0.033) and 0.49 (95% CI: 0.28–0.86, *P* = 0.012). The HR of patients with triple negative tumors was 0.50 (95% CI: 0.25–0.98, *P* = 0.043) and 0.45 (95% CI: 0.22–0.91, *P* = 0.026) (**Table 3**). The HR estimates among patients with these highly aggressive subtypes showed an association between NAC cycles and survival benefits after adjusting for age at diagnosis, clinical tumor stage, and post-NAC pathological node stage.

**Table 3:** Multivariate analysis for disease-free and overall survivals according to neoadjuvant chemotherapy cycles

NAC cycles	DFS			OS		
	Hazard ratio (95% CI)		*P*	Hazard ratio (95% CI)		*P*
Overall^§^						
Non-standard cycles	Ref	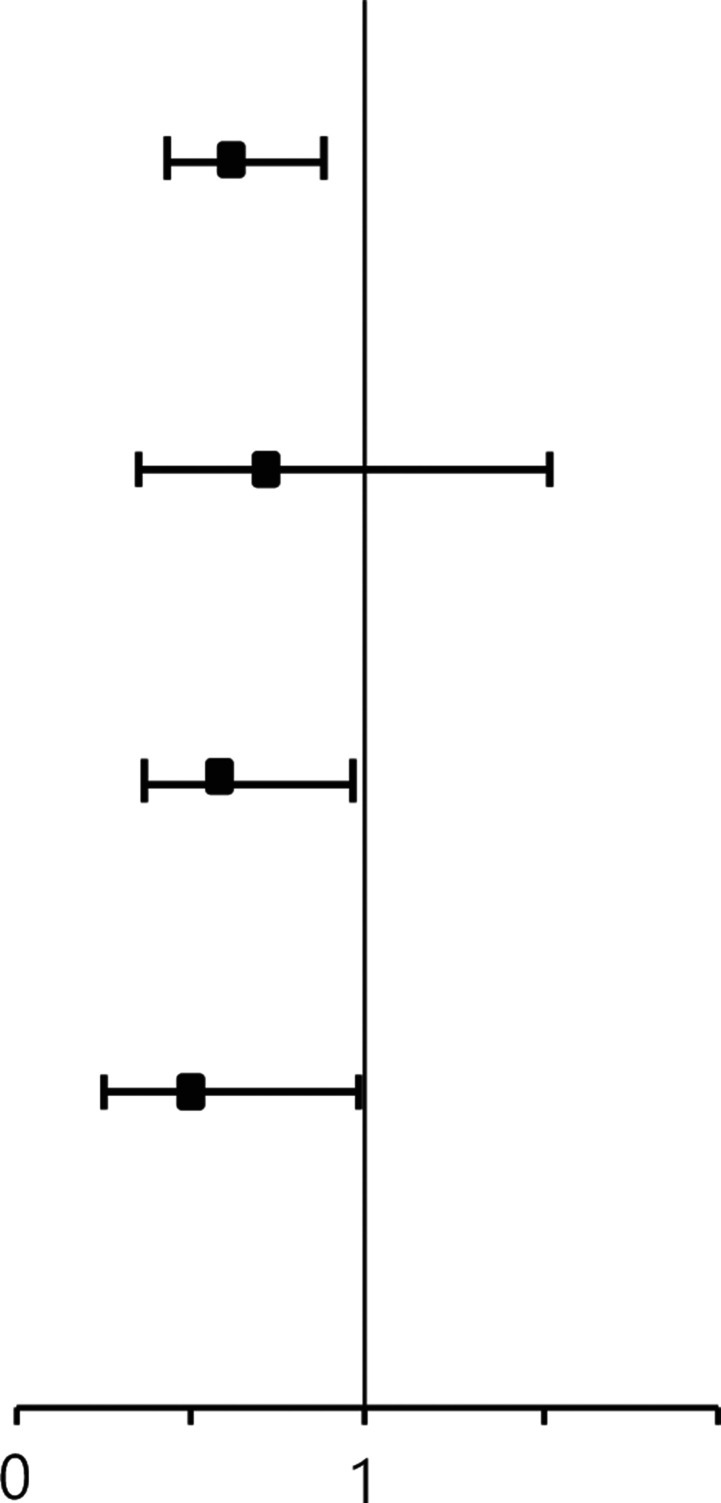		Ref	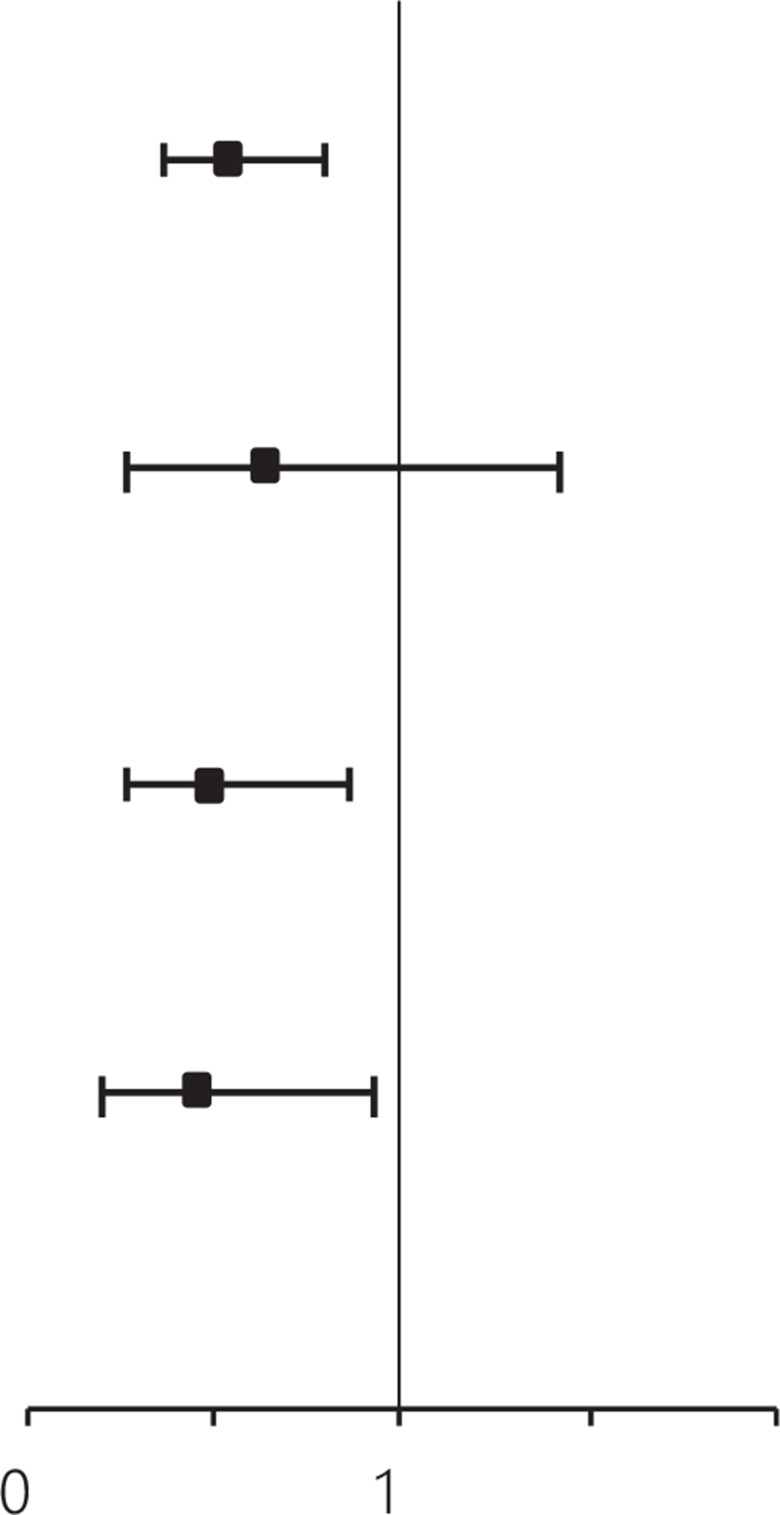	
Standard cycles	0.62 (0.44, 0.88)		0.007*	0.54 (0.37, 0.79)		0.001*
HR positive/HER2 negative^†^						
Non-standard cycles	Ref			Ref		
Standard cycles	0.72 (0.35, 1.52)		0.393	0.63 (0.29, 1.40)		0.255
HER2 positive^†^						
Non-standard cycles	Ref			Ref		
Standard cycles	0.59 (0.36, 0.96)		0.033*	0.49 (0.28, 0.86)		0.012*
Triple negative^†^						
Non-standard cycles	Ref			Ref		
Standard cycles	0.50 (0.25, 0.98)		0.043*	0.45 (0.22, 0.91)		0.026*

*P < 0.05 was considered statistically significant.

^§^Adjusted for neoadjuvant chemotherapy cycles, age at diagnosis, clinical tumor stage, biological subtypes, and post-neoadjuvant pathological node stage.

^†^Adjusted for neoadjuvant chemotherapy cycles, age at diagnosis, clinical tumor stage, and post-neoadjuvant pathological node stage.

HR, hormone receptor; HER2, human epidermal growth factor receptor-2; pCR, pathological complete response; NAC, neoadjuvant chemotherapy; DFS, disease-free survival; OS, overall survival.

**Figure 3:**
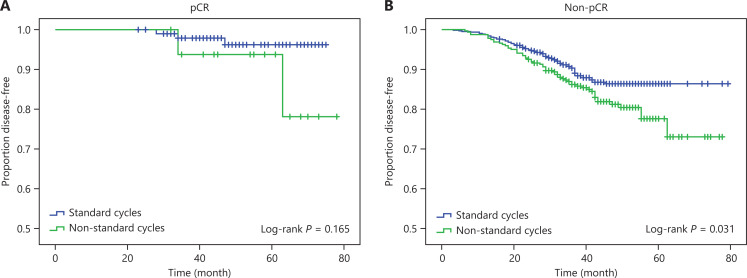
The Kaplan-Meier method estimated disease-free survival by standard and non-standard neoadjuvant chemotherapy cycles, in patients with pathological complete response (pCR) (A), and patients with non-pCR (B).

**Figure 4:**
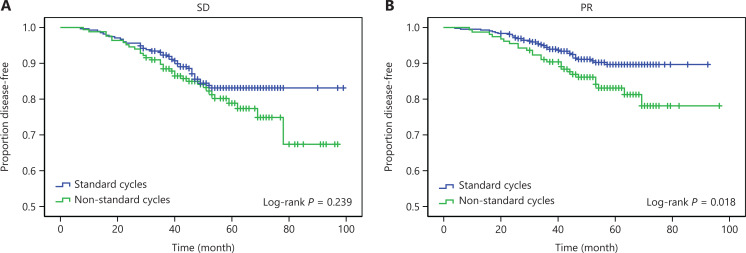
The Kaplan-Meier method estimated disease-free survival by standard and non-standard neoadjuvant chemotherapy cycles, in patients with stable disease (A), or patients with partial response (B).

Subgroup analyses according to neoadjuvant pathological response and clinical response are shown in **Figures 3, 4 and Supplementary Figures S3 and S4**. No significant survival benefits were observed in the standard NAC group among patients with pCR after NAC (**Figure 3A and Supplementary Figure S3A**). Treatment with non-standard NAC cycles had a significant detrimental effect on patients without pCR, with 5-year DFS percentages of 80.3% and 85.7% (*P* = 0.031) (**Figure 3B**) and 5-year OS percentages of 82.0% and 88.9% (*P* = 0.008) (**Supplementary Figure S3B**) for the non-standard and standard NAC groups, respectively. **Figure 4 and Supplementary Figure S4** show that considering the effects of neoadjuvant clinical response, patients with PR might benefit more from standard NAC cycles than from non-standard cycles (DFS *P* = 0.018 and OS *P* = 0.005); however, the relationship between NAC cycles and survival among patients with SD was not statistically significant.

## Discussion

To determine the importance of standard NAC treatment cycles, we conducted a study involving 1,024 patients who were diagnosed with invasive primary breast cancer and treated with at least 1 cycle of NAC followed by surgery. We found that standard NAC cycles were clearly associated with favorable long-term survival outcomes, and the treatment cycle regimen could act as an independent prognostic factor for patients with breast cancer. The results of our study are consistent with previous results, such as those from the ABCSG trial showing that doubling the number of cycles of neoadjuvant epirubicin, docetaxel, and granulocyte colony-stimulating factor (ED+G) from 3 to 6 cycles may result in higher rates of pCR^14^. The association between NAC cycles and long-term outcomes was significantly evident in patients with HER2 positive and triple negative subtypes in our subgroup analysis. Overall, our results showed the feasibility of using clinical and pathological response-guided approaches.

Norton-Simon et al.^15^ previously proposed a dose intensive hypothesis to effectively control the regrowth of tumor cells by increasing the frequency of drug administration based on the Gompertzian model. Subsequently, the Goldie-Coldman hypothesis further showed that dose-intensive administration could prevent the development of drug-resistant mutant clones^16^. Shortening the administration interval time allowed for continuous drug exposure, promoting apoptosis and anti-angiogenesis, thereby achieving the maximum tumor cell killing effect and improving the likelihood of achieving a cure^17^. These dose-intensive hypotheses were evaluated in clinical trials, and the NCCN guidelines were rewritten based on the results of the CALGB 9,741 trial^18^. The dose-intensive regimen containing anthracycline- and/or taxane-based chemotherapy is considered to be the preferred option in both neoadjuvant and adjuvant settings, especially for patients with high risk breast cancer. The Early Breast Cancer Trialists’ Collaborative Group (EBCTCG) report on a patient-level meta-analysis of 37,298 women with early-stage breast cancer further confirmed that increasing the dose intensity of adjuvant chemotherapy by more frequent administration or sequential scheduling might improve outcomes^19^. Based on the results of our study, we recommend that standard chemotherapy cycles should be given as a time-intensive strategy prior to surgery rather than separated into neoadjuvant and adjuvant phases for patients with HER2 positive and triple negative disease.

Previous studies have indicated that pCR was correlated with improved DFS and OS^4^. The pCR is a potential surrogate endpoint in neoadjuvant settings for HER2 positive and triple negative disease^5^. Our findings showed a significant association between pCR and long-term outcomes in patients with triple negative subtypes. Furthermore, recent studies have highlighted the applicability of pCR for risk stratification and the selection of subsequent adjuvant treatments. In the CREATE-X trial, the use of capecitabine in patients with HER2 negative disease without pCR following at least 4 cycles of NAC conferred a significant survival advantage in the triple negative cohort^6^. In the KATHERINE trial, the use of trastuzumab emtansine (T-DM1) among patients with HER2 positive residual tumors after at least 6 cycles of NAC was shown to reduce the risk of recurrence and death by nearly 50%^7^. Thus, the addition of non-cross-resistant adjuvant strategies was shown to be valuable, and the clinical utility of therapeutic escalation approaches based on NAC response was confirmed. Additionally, the assessment of pathological response based on a complete NAC cycle was shown to be consequential, and it affirmed the necessity of standard NAC treatment cycles. Survival of patients without pCR was significantly better in patients receiving standard NAC cycles than in patients receiving non-standard NAC cycles in our study.

The similar outcomes seen in patients achieving pCR after NAC with or without adjuvant chemotherapy suggested that adjuvant chemotherapy could potentially be omitted in certain circumstances. A meta-analysis at the San Antonio Breast Cancer Symposium in 2018 found similar survival in patients with pCR followed by adjuvant chemotherapy [5-year event-free survival (EFS): 86%], and those without additional adjuvant chemotherapy (5-year EFS: 88%). Similarly, the results of the WSG-ADAPT-TN trial indicated that the criterion of pCR after 12 weeks of the anthracycline-free NAC regimen could be used for treatment de-escalation decisions in patients with triple negative tumors^8^. We found that NAC cycles were not significantly associated with survival in patients with pCR, which is consistent with the concept of de-escalation strategies. The de-escalation of further adjuvant chemotherapies in patients with pCR following a neoadjuvant regimen might be a promising topic for future research. We expect further investigations, such as the COMPASS trial to assess the clinical utility of escalation or de-escalation protocols in the adjuvant setting based on the neoadjuvant response.

Finally, the neoadjuvant approach offers opportunities for response-guided therapeutic strategies, whereby therapeutic regimens can be adjusted when tumor tissue is available for response monitoring. It is well-established that patients suffering from PD during NAC should be given an alternate treatment or shifted to surgery. The choice of treatment regimens and the timing of surgery in the neoadjuvant period for patients with PR or SD remain controversial. The GeparTrio trial showed that DFS could be improved by prolonged chemotherapy for early responders and by switching nonresponders to a non-cross-resistant regimen, especially in patients with HR positive tumors. However, the results did not support modifying chemotherapy regimens in patients with HER2 positive or triple negative disease^20^. Our results indicated that patients with PR attained better survival outcomes with standard NAC cycles, so we suggest that patients with breast cancer should complete at least a 4-cycle standard NAC regimen. However, as there was no significant survival benefit in patients with SD, continuation or switching approaches might be reasonable choices that require further investigation.

It should be noted that this was a study of a hospital-based database, which is one of the limitations of this study. We acknowledge the limitations associated with the retrospective nature of our study. The percentage of patients with pCR was relatively small, with 11.3% of overall patients, 15.2% of patients with triple negative, and 12.7% of patients with HER2 positive disease achieving pCR, mainly because patients enrolled in clinical trials were not always representative of the general population, and there may be a discordance between chemotherapy strategies used in medical practice conditions with those used in a more skilled practice. It is possible that more recurrence and death events would have been identified with a longer follow-up period, particularly among patients with HR positive disease. We also had no detailed information regarding residual potential confounders such as the time interval of treatment or chemotherapy regimen dosage, nor could we identify reasons why patients discontinued standard NAC cycles. Furthermore, while prospective data could provide high quality evidence to answer clinical questions, a prospective randomized trial will likely never be conducted on this issue due to the ethical aspects. Finally, molecular profiling could provide valuable individualized information for patients and aid physicians in stratifying patients to make more suitable treatment decisions. There is an increasing need for evidence to bridge the gap between clinical trials and clinical practice, which prompted the design and conduct of this study.

## Conclusions

Our study indicated that treatment with standard NAC cycles provided a significant survival benefit for breast cancer patients, especially for patients with HER2 positive and triple negative cancers. Standard NAC regimens with at least 4 cycles should be provided before surgery, rather than splitting cycles into preoperative and postoperative phases. We further highlighted the clinical effectiveness of escalation or de-escalation therapeutic approaches based on neoadjuvant responses. We expect further investigations to focus on considering NAC cycles to formulate treatment recommendations for patients with breast cancer in an organized and efficient manner.
