# Supplementary material for: The choice of a neoadjuvant chemotherapy cycle for breast cancer has significance in clinical practice: results from a population-based, real world study
Source: Cancer Biol Med. 2021 Oct 12;19(5):755–67. doi: 10.20892/j.issn.2095-3941.2020.0800 (PMC9196050; doi:10.20892/j.issn.2095-3941.2020.0800)
Supplement: Supplementary file 1 [file cbm-19-755-s001.pdf]

Supplementary materials

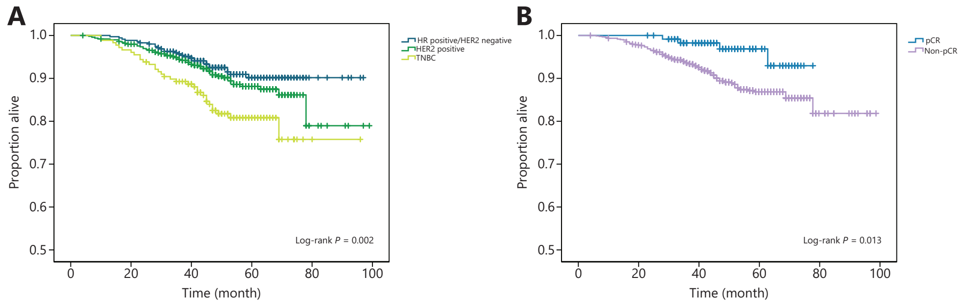

Figure S1 The Kaplan-Meier method estimated overall survival by biological subtypes (A) and pathological response (B).

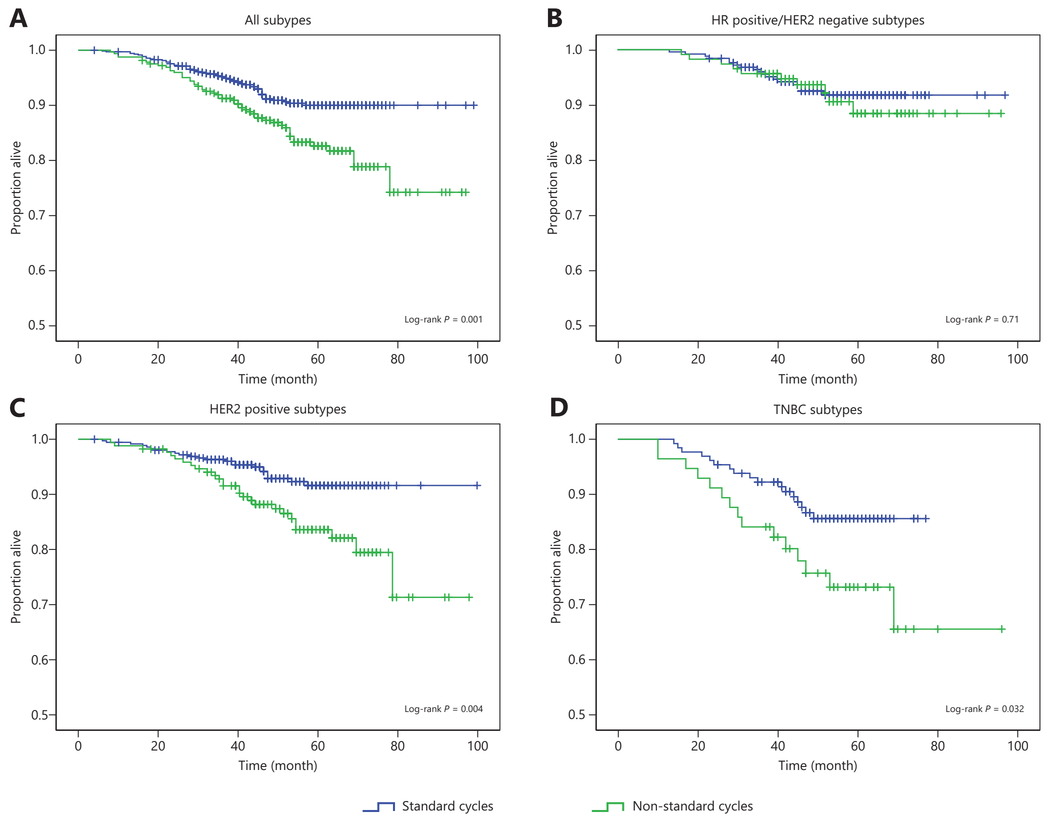

Figure S2 The Kaplan-Meier method estimated overall survival by standard and non-standard neoadjuvant chemotherapy cycles in overall subtypes (A), HR positive/HER2 negative subtypes (B), HER2 positive subtypes (C), triple negative subtypes (D).

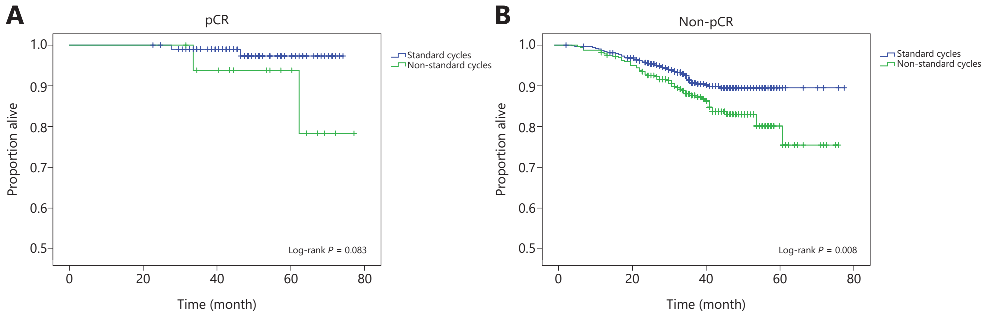

Figure S3 The Kaplan-Meier method estimated overall survival by standard and non-standard neoadjuvant chemotherapy cycles, in patients with pathological complete response (pCR) (A), and patients with non-pCR (B).

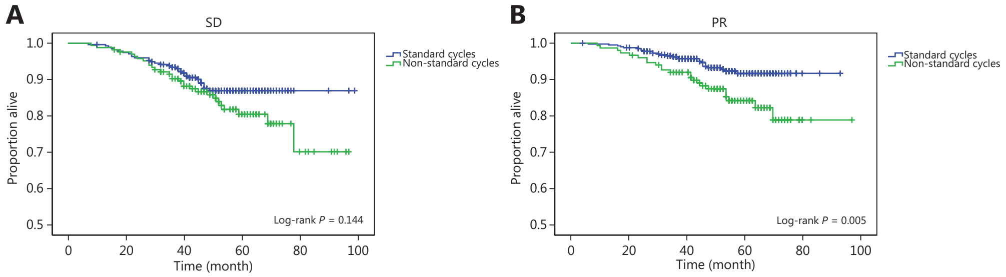

Figure S4 The Kaplan-Meier method estimated overall survival by standard and non-standard neoadjuvant chemotherapy cycles, in patients with stable disease (A), patients with partial response (B).

Table S1 The association between pathological response and long-term outcomes by tumor biological subtypes

Pathological response	Total	DFS		OS	
		Events	Log-rank *P* value	Events	Log-rank *P* value
Overall					
pCR	116	5	**0.006***	4	**0.013***
Non-pCR	908	131		107	
HR positive/HER2 negative					
pCR	26	0	0.11	0	0.148
Non-pCR	325	34		28	
HER2 positive					
pCR	63	4	0.13	3	0.185
Non-pCR	432	63		48	
Triple negative					
pCR	27	1	**0.031***	1	**0.043***
Non-pCR	151	34		31	

*P < 0.05 was considered statistically significant.
HR, hormone receptor; HER2, human epidermal growth factor receptor-2; pCR, pathological complete response; DFS, disease-free survival; OS, overall survival.

Table S2 Multivariate analyses for disease-free and overall survivals

	DFS		OS	
	Hazard ratio (95% CI)	*P* value	Hazard ratio (95% CI)	*P* value
Age at diagnosis	1.01 (0.99, 1.02)	0.590	1.00 (0.98, 1.02)	0.841
Clinical tumor stage				
T1	Ref		Ref	
T2	0.99 (0.55, 1.77)	0.961	0.93 (0.49, 1.77)	0.832
T3/T4	1.93 (1.02, 3.64)	**0.044***	2.04 (1.02, 4.07)	**0.044***
Biological subtype				
HR positive/HER2 negative	Ref		Ref	
HER2 positive	1.53 (1.01, 2.32)	**0.047***	1.38 (0.86, 2.20)	0.179
Triple negative	2.55 (1.58, 4.10)	**< 0.0001***	2.81 (1.68, 4.69)	**< 0.0001***
Post-neoadjuvant pathological node stage				
ypN0	Ref		Ref	
ypN1	1.52 (0.93, 2.48)	0.093	1.47 (0.85, 2.54)	0.17
ypN2	3.45 (2.16, 5.51)	**< 0.0001***	3.39 (2.01, 5.71)	**< 0.0001***
ypN3	3.22 (1.96, 5.28)	**< 0.0001***	3.41 (1.98, 5.85)	**< 0.0001***
NAC cycles				
Non-standard cycles	Ref		Ref	
Standard cycles	0.62 (0.44, 0.88)	**0.007***	0.54 (0.37, 0.79)	**0.001***

*$P < 0.05$ was considered statistically significant.
ER, estrogen receptor; PgR, progesterone receptor; HER2, human epidermal growth factor receptor-2; HR, hormone receptor; pCR, pathological complete response; NAC, neoadjuvant chemotherapy.
